# A reference-defining criterion for light focusing through scattering media based on circular Gaussian distribution of speckle background intensity

**DOI:** 10.1038/s41598-018-24698-0

**Published:** 2018-04-23

**Authors:** Bin Zhang, Zhenfeng Zhang, Qi Feng, Chengyou Lin, Yingchun Ding

**Affiliations:** Department of Physics, Beijing University of Chemical Technology, Beijing, 100029 China

## Abstract

This paper investigates the reference-defining-criterion problem in the field of light focusing through scattering media. In many analogous light focusing experiments, the enhancement values differ greatly from each other. By analyzing the focusing picture after optimizations, we concluded that the discrepancy in enhancement originates from the unclear definition of reference boundary. By averaging multiple speckle backgrounds, we found that the intensity of speckle background obeys circular Gaussian distribution. Based on the intensity statistics and Gaussian-function fitting to the speckle background, we proposed a clear reference-defining criterion– 1/*e*^2^ criterion. With this reference-defining criterion, we have carried out light focusing experiments with the speckle backgrounds possessing different shape and size. The enhancements obtained from the repetitive experiments for both weakly scattering medium and strongly scattering medium were all in the reasonable range, demonstrating its validity and universality. This criterion will provide a comparison standard for light focusing experiments in wavefront-shaping field.

## Introduction

Spatial inhomogeneities in the refractive index of random photonic materials such as paper, paint, and biological tissue cause multiply scattering of light. As a result, light propagates diffusively, which makes the control of light become impossible with conventional optics. However, it has recently been demonstrated that light can be controlled by shaping the wavefront of incident light^1^. With this technique, we can achieve light focusing through scattering media, which plays an important role in biomedical imaging and therapy^2–4^. In 2007, Vellekoop *et al*.^3^ firstly realized light focusing through scattering media with the iterative optimization method, which is also the first demonstration of wavefront-shaping technique^5^. Nowadays, the light focusing wavefront-shaping techniques can be divided into three categories: iterative optimization method^6^, transmission matrix method^7^, and phase conjugation method^8^. The research contents of this paper are based on the iterative optimization method.

The primary elements of the iterative optimization method are the optimization algorithms and light modulators. In the iterative optimization method, the optimization algorithm controls the modulator to modulate the phase or amplitude of the incident light based on a feedback signal to perform iterative optimizations until finding the optimal mask for creating a focus^5^. Currently, the existing optimization algorithms can be divided into two categories: general algorithms and intelligent algorithms. Intelligent algorithms show better noise-resistance capability than the general algorithms such as stepwise sequential algorithm, continuous sequential algorithm, and partitioning algorithm^6,9^. In 2012, Conkey *et al*.^10^ introduced the genetic algorithm (a typical intelligent algorithm) into the wavefront-shaping field and demonstrated its strong noise-resistance capability^10^. In the aspect of light modulator, according to the form of light modulation, the existing light modulators can be divided into two categories: phase modulators and amplitude modulators. The representative devices are liquid-crystal-based spatial light modulation (SLM) and digital mircomirror device (DMD) respectively. SLMs show better light focusing performance, while DMDs show faster modulation speed^11^. The light focusing experiments performed in this paper are based on the amplitude-only genetic algorithm with the DMD.

Enhancement is a vital figure of merit describing the effectiveness of a light focusing experiment. It is defined as the ratio of focus intensity to reference intensity: *η* ≡ *I*_*opt*_/*I*_*ref*_^9^. Where *I*_*opt*_ is the average intensity in the focus after optimizing the incident wavefront, and *I*_*ref*_ is the average intensity of the reference. In a strongly scattering medium, the translation paths are statistically independent and follow a circular Gaussian distribution. In this case, the maximum enhancement that can be achieved is *η* = *α* (*N* + 1) + 1^12^, where *N* is the number of independently controlled segments of the incident light, and *α* is the relative enhancement. The value of *α* depends on the type of light modulation that is used^13^. In the past work, for an amplitude-only modulation system, the numbers of the opened and closed segments were thought to be approximate equal in the optimal amplitude masks, and the target position intensity (TPI) was used as the discriminant in the optimization algorithms.Under this circumstance, the value of *α* was deduced to be 1/(2*π*) ≈ 15.9%^14^. In 2017, Feng *et al*.^15^ demonstrated a new discriminant–signal to background ratio (SBR) discriminant. According to their numerical simulation results, the value of *α* can be as high as 22.5%^15^. However, in many analogous amplitude-optimization experiments with the TPI discriminant or the SBR discriminant, the practical enhancements were rarely able to achieve the theoretical maximum or value of simulation. Furthermore, the enhancement values differ greatly from each other^14,16^. Indeed, the noise of different experimental systems is one reason for the discrepancy in enhancement^14^. However, we now encounter another problem that cannot be ignored–the ambiguity definition of the reference^12,17^. Though Vellekoop and Akbulut demonstrated the specific determining method of the reference respectively^9,14^, they did not indicate the boundary of the reference, which determines directly the value of enhancement.

In this paper, we have demonstrated the discovery of the reference-defining problem in the experiment of focusing light through scattering media using a DMD. After sound analysis to the speckle background, we concluded that the selected radius of the speckle background had a direct influence on the value of enhancement. Based on the study of the intensity distribution of speckle background, we proposed a clear reference-defining criterion– 1/*e*^2^ criterion. With this criterion, we have carried out repetitive light focusing experiments with speckle backgrounds possessing different shape and size. The enhancements obtained were all slightly below the value of simulation or theoretical maximum, demonstrating its validity and universality. This 1/*e*^2^ criterion is applicable and valid in defining the reference in the whole field of light focusing through scattering media. Therefore, it will provide a comparison standard for light focusing experiments in wavefront-shaping field.

## Experiment

**Figure 1:**
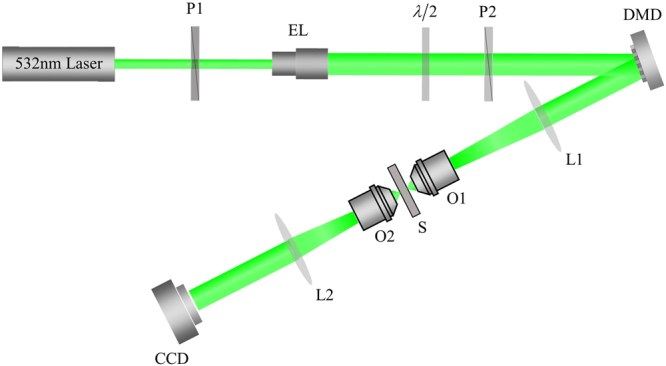
Experimental setup. P1, P2, polarizers; EL, expending lens; *λ*/2, half wave plate; DMD, digital micromirror device; L1, L2, lenses with focal length of 150 and 50 mm respectively; O1, 40× objective with 0.65 numerical aperture; S, sample; O2, 10× objective with 0.25 numerical aperture.

The schematic layout of the experimental setup is shown in Fig. 1. A 532-nm semiconductor laser with output power of 10 mW is used as the light source. The polarizer changes the polarization state of the incident light from elliptic polarization to linear polarization. Then the output beam from P1 is expanded by an expending lens to illuminate the entire area of the DMD (Texas Instruments DLP 6500, 1920 × 1080 pixels). The *λ*/2 wave plate and P2 are used to change the light intensity that illuminates on the DMD without changing the polarization state of the incident light. The wavefront of the reflected beam is shaped by selectively turning the mircomirrrors on and off. The modulated beam is focused on the sample by a 40 × objective. Then the transmitted light is collected by a 10 × objective and monitored by a CCD camera (AVT Manta G-031B, 656 × 492 pixels, pixel size 5.6 *μm* × 5.6 *μm*). The scattering sample used in the experiment is the ground glass diffuser (Edmund # 45–653).

The iterative algorithm we used to focus light through scattering media was genetic algorithm. We divided the selected 160,000 DMD pixels into 400 controllable segments. Every segment can be controlled independently by the DMD. The amplitude masks generated by the genetic algorithm were projected on the DMD. The CCD camera captured the corresponding speckle patterns and sent them back to the algorithm. The running environment of the genetic algorithm was Matlab 2014b. Besides, the fitness function of the genetic algorithm we used was the SBR discriminant, which reveals better focusing performance than the traditional TPI discriminant^15^.

**Figure 2:**
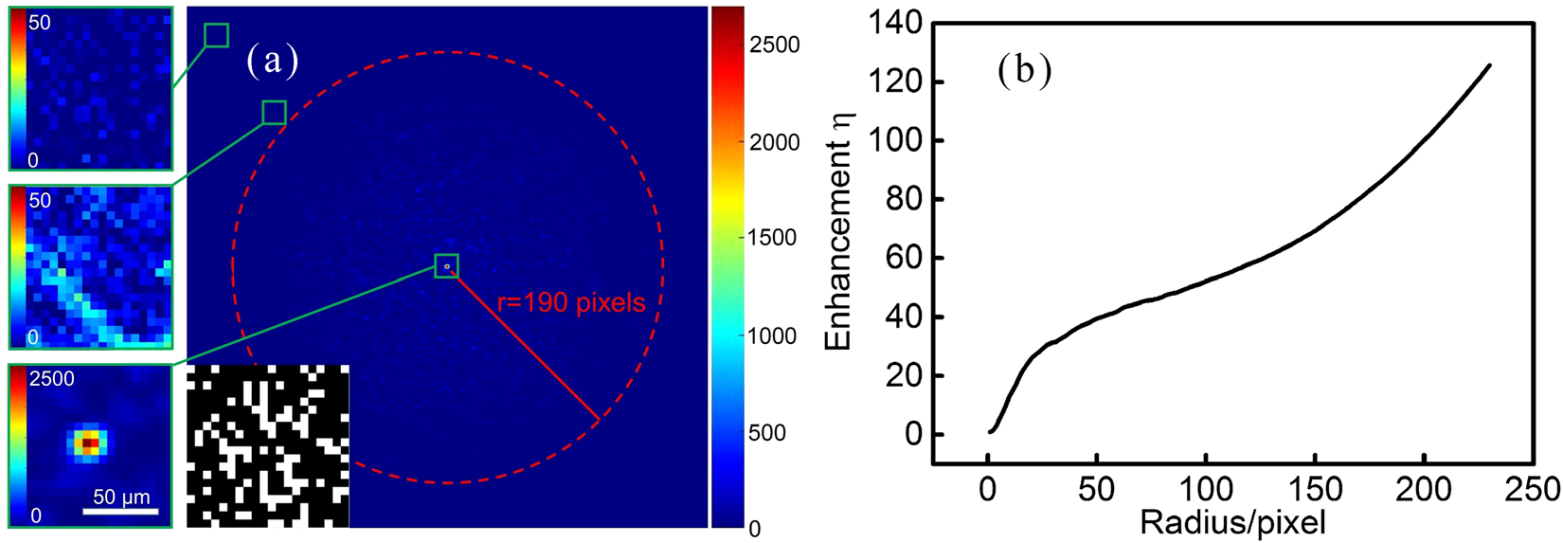
Experimental results of single-spot focusing through the ground glass diffuser. (**a**) Focusing picture captured by the CCD. r = 190 pixels is the background radius selected in the experiment. The related parameters of the genetic algorithm: population 50, generation 250, segments number 400, crossover probability 0.9, mutation probability 0.002. It takes 945 s to complete the 6300 iterations, which is limited by the picture acquisition time of the CCD camera. (**b**) The variation of enhancement with different reference radiuses. This enhancement curve derives from the focusing picture in (**a**). Strictly speaking, this is an approximate solution of the actual experiments with different radiuses, since the focus intensities for the experiments with different radiuses are slightly different.

In this experiment, we chose 3 × 3 pixels as the target area and set the background radius as 190 pixels. After 6300 iterations, the genetic algorithm arrived at an optimal amplitude mask, which is shown in the lower left corner of Fig. 2(a). Loading this optimal amplitude mask on the DMD, we got a bright focus with an enhancement of 90 in the middle of the speckle pattern, shown in Fig. 2(a). The inserts present the magnification for different areas in the focusing picture. The diameter of the focus is about 15 *μm* (see the nethermost insert).

Having a close-up view of the focusing picture (see the two upper inserts), one can find that the area that is just out of the 190-pixels radius still possesses nonzero light intensity. According to the focusing picture in Fig. 2(a), we made a drawing of the enhancement curve with variable reference radius, shown in Fig. 2(b). One can see that the enhancement increases with the increscent reference radius. Furthermore, when the radius is larger than 192 pixels, the enhancement exceeds the value of 90.8, which is the value of simulation for the SBR discriminant with 400 light segments^14^. Thus, we have got an “unreasonable” experimental result. Figure 2(b) is obtained from Fig. 2(a), where the intensity in the focus is a constant. Therefore, the varying value of enhancement is due to the varying reference.

## 1/***e***^2^ criterion

**Figure 3:**
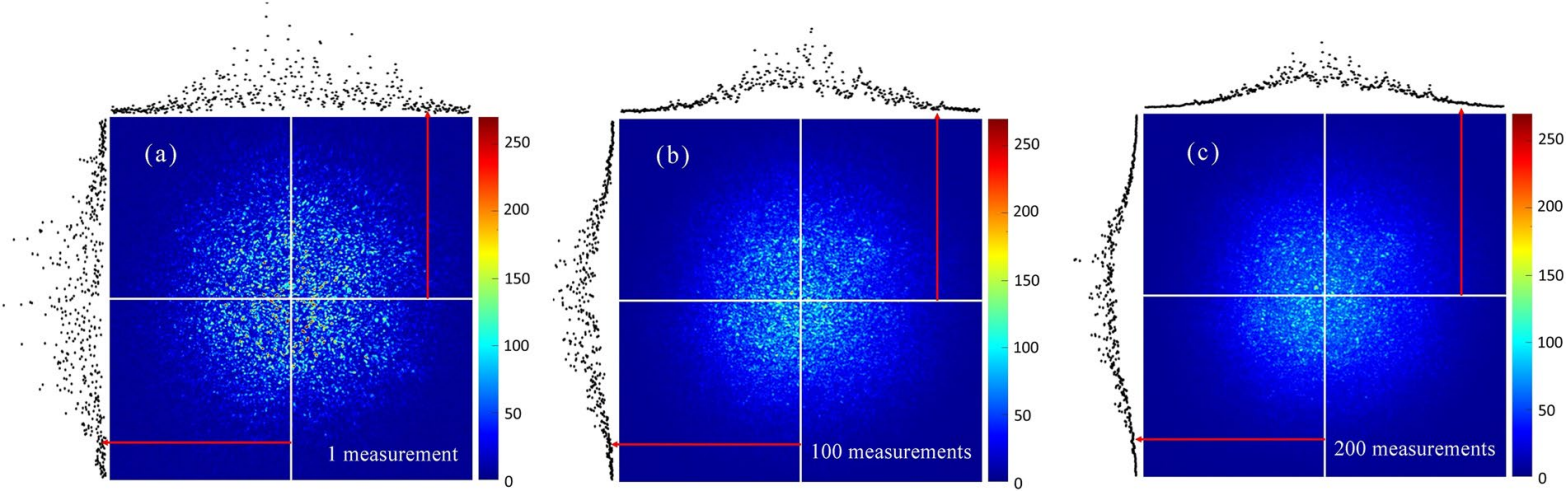
The intensity distribution characteristic of the speckle background. (**a**) A speckle background and its intensity distribution for a random amplitude mask. (**b**,**c**) are the averaged speckle patterns for 100 and 200 measurements respectively. The black spots shows the gray values of the pixels along the white lines.

The intensity distribution of the reference in the focusing picture is similar to the intensity distribution of the speckle background before optimization. So we can analyze the distribution of reference from the angle of speckle background. Figure 3(a) shows a typical picture of speckle background with a random amplitude mask. Note that the gray values reflect the intensity of the light detected by the CCD camera. From Fig. 3(a), we can not find an explicit distribution. In consideration of that speckles originate from the light-interference process possessing randomness characteristics, we have tried to study the statistical distribution of speckle background intensity. We first loaded multiple amplitude masks on the DMD and captured the corresponding speckle patterns. Then, we superposed the multiple speckle patterns and took the average. Figure 3(b) and (c) are two averaged speckle patterns for 100 and 200 measurements respectively. We can find that, with 200 measurements, the intensity distribution of the speckle background approaches Gaussian distribution. Because the speckle background is circular, the intensity distribution along an arbitrary line passing through the centre of the speckle background follows Gaussian distribution as well. Thus, we can conclude that the intensity of speckle background follows circular Gaussian distribution.

Inspired by the definition of Gaussian beam radius, we have tried to take a radius where the intensity is 1/*e*^2^ the maximum of the Gaussian function. In consideration of the circular-Gaussian-distribution characteristic, to obtain an accurate statistical distribution of the speckle background intensity, we have designed the following steps. First, we loaded 200 random masks on the DMD and recorded the corresponding speckle patterns on the CCD camera. Then we superposed the 200 speckle patterns and took the average. Thus, an averaged speckle background was obtained. Second, with the averaged speckle background, we drew a series of circles about the background centre. Then the mean gray value of the pixels on each circle was calculated. In this way, we got a homogeneous gray value for every circle. Third, we plotted the mean gray values for different radiuses and made a Gaussian-function fitting on them with the Gaussian-curve-fitting tool in the Matlab software.

**Figure 4:**
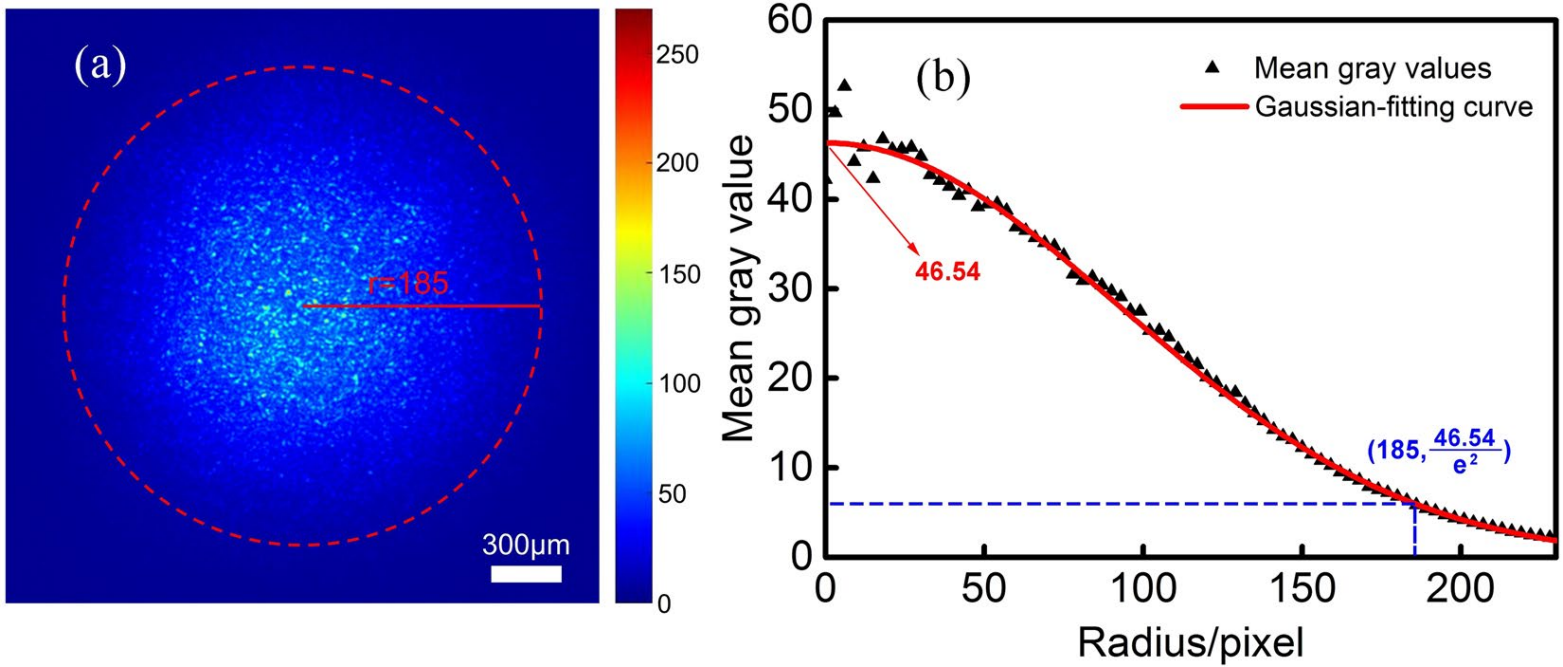
The averaged speckle background and the corresponding Gaussian-function-fitting result for the light focusing experiment demonstrated in Section 2. (**a**) The averaged speckle background for 200 measurements. (**b**) The intensity distribution for the averaged speckle background and the fitting result. The black triangles are the mean gray values for different radiuses, and the red line is the Gaussian-function-fitting curve on them.

The data processing result is shown in Fig. 4. Figure 4(a) is the averaged speckle background for the experiment demonstrated in Section 2. The mean gray values of the pixels for different radiuses are presented as the black triangles, and the corresponding Gaussian-function-fitting curve is shown as the red line in Fig. 4(b). One can see that the mean gray values fit quite well with the fitting curve. Note that, in view of the circle-drawing operation in step 2, we only got “half” of the intensity distribution curve. The Gaussian function obtained here was *I*(*r*) = 46.54 exp (−*r*^2^/130.7^2^), whose maximum value was 46.54. Then, we chose a radius where the mean gray value is 46.54/*e*^2^. In this way, this radius was calculated to be approximately equal to 185 pixels. The radius used to demarcate the reference in Fig. 2 was 190 pixels, which leaded to an enhancement value of 90. According to the enhancement curve demonstrated in Fig. 2(b), we calculated that, with the 185-pixels radius, the enhancement should be 89.3. The enhancement value of 89.3 is in the reasonable range (below the value of simulation). So we made an assumption of the 1/*e*^2^ reference-defining criterion.

To further verify the validity of this 1/*e*^2^ reference-defining criterion, we have carried out the light focusing experiments with speckle backgrounds possessing different shape and size. The different speckle backgrounds were obtained by moving the ground glass diffuser slightly. In the iterative optimizations with the TPI discriminant, though *I*_*ref*_ is not calculated in each iteration, it is used to calculate the enhancement after optimization. Therefore, to test the universality of the 1/*e*^2^ criterion, except for the SBR discriminant, we have also used the TPI discriminant to perform the light focusing experiments.

**Figure 5:**
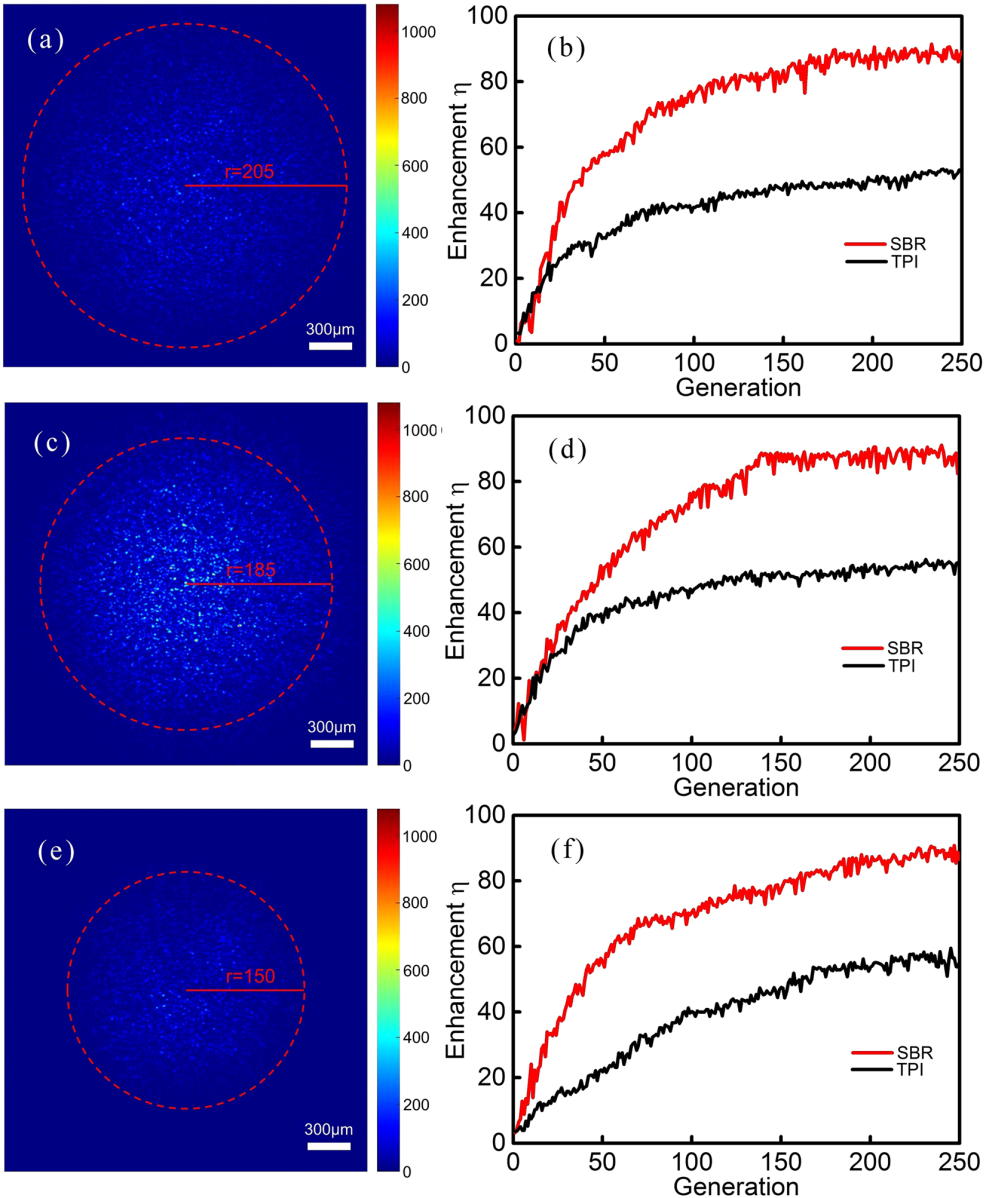
The iterative optimization results for different speckle patterns with the 1/*e*^2^ reference-defining criterion. (**a**,**c** and **e**) are the averaged speckle backgrounds with different shape and size. With our 1/*e*^2^ reference-defining criterion, the radiuses were confirmed to be 205, 185, and 150 pixels respectively. (**b**,**d** and **f**) are the typical optimization results for the speckle backgrounds in (**a**,**c** and **e**) respectively. The related parameters of the genetic algorithm: population 50, generation 250, segments number 400, crossover probability 0.9, mutation probability 0.002. The running time of this genetic algorithm is 945 s.

Figure 5(a),(c) and (e) are the averaged speckle backgrounds with different shape and size. With the 1/*e*^2^ reference-defining criterion, we confirmed that the radiuses of the speckle backgrounds were 205, 185, and 150 pixels respectively. For each speckle background, we performed 10 iterative optimizations–5 optimizations for the SBR discriminant and 5 optimizations for the TPI discriminant. Figure 5(b),(d) and (f) are the typical iterative optimization results for the different speckle backgrounds presented in Fig. 5(a),(c) and (e) respectively. With the SBR discriminant, the enhancements for the different speckle backgrounds are 88.4 ± 2.1 (sd), 86.6 ± 1.8 (sd), and 86.9 ± 2.3 (sd) respectively. And with the TPI discriminant, the enhancements are 52.3 ± 0.6 (sd), 54.1 ± 1.2 (sd), and 54.8 ± 2.2 (sd) respectively.

Except for the ground glass diffuser (weakly scattering medium), we have also carried out light focusing experiments with a 37 *μ*m-thick deposit of 200 nm ZnO nanoparticles (strongly scattering medium). The mean free path of this deposit was determined by measuring the total transmission and equals 0.96 ± 0.15 μm at a wavelength of 532 nm^12^. By suitably adjusting the positon of the ZnO sample, we have obtained the averaged speckle patterns with the radiuses of 203, 185, and 167 pixels respectively. Also, 5 optimizations for the SBR discriminant and 5 optimizations for the TPI discriminant were performed for each speckle background. To avoid repetition, we did not show the averaged speckle patterns and the evolutions of enhancement as shown in Fig. 5, but we give the values of enhancement. With the SBR discriminant, the enhancements for the different speckle backgrounds are 85.5 ± 1.1 (sd), 84.1 ± 2.2 (sd), and 86.3 ± 1.7 (sd) respectively. And with the TPI discriminant, the enhancements are 54.7 ± 1.2 (sd), 53.8 ± 2.0 (sd), and 53.7 ± 1.5 (sd) respectively.

In the amplitude-only optimization, the value of simulation of relative enhancement for the SBR discriminant is 22.5%^15^, and the theoretical maximum of the relative enhancement for the TPI discriminant is 15.9%^14^. Therefore, according to the maximum enhancement formula *η* = *α* (*N* − 1) + 1, for 400 light segments, the maximum enhancements for the SBR discriminant and the TPI discriminant are 90.8 and 64.5 respectively. The enhancements obtained from the testing experiments for both weakly scattering medium and strongly scattering medium were all in the reasonable range (below the value of simulation or theoretical maximum). Meanwhile the 1/*e*^2^ criterion is suitable for both SBR discriminant and TPI discriminant. In this way, the validity and universality of the 1/*e*^2^ reference-defining criterion have been verified.

## Conclusion

In this paper, we have carried out the experiments of focusing light through scattering media with genetic algorithm. By analyzing the focusing picture, we found that the enhancement obtained from the experiment was variable for different reference boundaries. Then we analyzed the speckle background and concluded that the unreasonable enhancements originates from the loophole in defining the reference. Inspired by the definition of Gaussian beam radius, we came up with a clear reference-defining criterion– 1/*e*^2^ criterion. With this 1/*e*^2^ criterion, we have carried out the light focusing experiments with the speckle backgrounds possessing different shape and size. Both the SBR discriminant and the TPI discriminant were used respectively in the experiments. The enhancements obtained from the experiments for both weakly scattering medium and strongly scattering medium were all in the reasonable range. Thus, the validity and universality of the 1/*e*^2^ criterion were verified. Besides, in phase-only modulation systems, the definition of enhancement involves the reference-defining problem as well. So the 1/*e*^2^ criterion can also be used as a criterion in phase-only modulation systems. In summary, the 1/*e*^2^ criterion is applicable and valid in both amplitude-only modulation and phase-only modulation systems. It will provide a comparison standard for light focusing experiments in wavefront-shaping field.
